# Early prediction model for prognosis of patients with hepatitis-B-virus-related acute-on-chronic liver failure received glucocorticoid therapy

**DOI:** 10.1186/s40001-022-00891-w

**Published:** 2022-11-14

**Authors:** Shuai Gao, Li-Yan Han, Yu-Chen Fan, Kai Wang

**Affiliations:** 1 Department of Hepatology, Qilu Hospital, Cheeloo College of Medicine, Shandong University, Jinan, Shandong China; 2 Hepatology Institute of Shandong University, Jinan, Shandong China

**Keywords:** Acute-on-chronic liver failure, Glucocorticoid, Noninvasive model, Prognosis, Hepatitis B virus

## Abstract

**Background:**

Early prediction for short-term prognosis is essential for the management of hepatitis B virus (HBV)-related acute-on-chronic liver failure (ACLF). In this study, we aim to establish a noninvasive model for predicting the 90-day mortality in patients with HBV–ACLF received glucocorticoid therapy.

**Methods:**

Two hundred and eighty patients with HBV–ACLF were enrolled from July 2010 to June 2022. All patients received routine medicine treatment and 204 of them received additional glucocorticoid treatment. Then, the patients who received glucocorticoid treatment were randomly divided into a training cohort and a validation cohort. An early prediction model for 90-day mortality of HBV–ACLF was established in the training cohort and then validated in the validation cohort.

**Results:**

HBV–ACLF patients received glucocorticoid treatment showed significantly better survival that those not (*P* < 0.01). In the training cohort, a noninvasive model was generated with hepatic encephalopathy grade, INR, total bilirubin, age and SIRS status, which was named HITAS score. It showed significantly better predictive value for 90-day mortality of HBV–ACLF than MELD score and Child–Turcotte–Pugh score in both the training cohort and validation cohort. Using the Kaplan–Meier analysis with cutoff points of 2.5 and 3.47, the HITAS score can classify HBV–ACLF patients into different groups with low, intermediate and high risk of death after glucocorticoid therapy.

**Conclusions:**

We proposed a HITAS score, which was an early prediction model for the prognosis of HBV–ACLF. It might be used to identify HBV–ACLF patients with favorable responses to glucocorticoid treatment.

## Introduction

Acute-on-chronic liver failure (ACLF) is a life-threatening clinical syndrome and occurs during an acute insult in the patients with chronic liver diseases, which is manifested as jaundice, coagulopathy, ascites as well as encephalopathy [1]. In Asia, hepatitis B virus (HBV) infection is the main cause of ACLF, which is named HBV–ACLF. The short-term mortality of HBV–ACLF is as high as 63–72.3% [2]. Until now, liver transplantation remains the determined cure for patients with HBV–ACLF who cannot be improved after supportive measures [3]. However, the application of liver transplantation is challenged by the lack of donors in most countries [4]. Therefore, great efforts have been made for finding new therapeutic and supportive approaches to HBV–ACLF.

Immunologic imbalance plays a vital role in the pathogenesis of HBV–ACLF. In patients with HBV–ACLF, liver injury is mainly induced by cytotoxic T-lymphocyte mediated damage in HBV-infected hepatocytes [5]. Glucocorticoids can suppress exaggerated immune responses and are widely used in the treatment of many diseases. Recent studies found that glucocorticoids could also prevent immunologic injury in infected hepatocytes and might be effective in the treatment of HBV–ACLF [6, 7]. However, glucocorticoids also have side effects and only a part of the HBV–ACLF patients can benefit from them. Until now, it is still difficult to predict the HBV–ACLF patients with favorable responses to glucocorticoids treatment from those not. Therefore, there is an urgent need for early prognostic models to stratify the death risks in patients with HBV–ACLF received glucocorticoid treatment.

In previous studies, several efforts have been made to find out indicators for predicting the treatment efficacy of glucocorticoids in patients with ACLF. A previous study found that patients responding best to glucocorticoids treatment were those with a lower MELD score and hepatic encephalopathy (HE) grade but extremely high alanine aminotransferase (ALT) levels [7]. Zhao et al. demonstrated that higher level of myeloid dendritic cells (mDCs) at baseline and a continuous increase in mDC numbers might predict a favorable response to glucocorticoid treatment [8]. In our previous study, we found that SOCS1 methylation was a good prognostic biomarker for patients with HBV–ACLF received glucocorticoid treatment [9]. Another study of us showed that HBV–ACLF patients with low levels of Tβ4 methylation might have a favorable response to glucocorticoid treatment [10].

In this study, we attempt to establish a noninvasive model for predicting the 90-day mortality in patients with HBV–ACLF received glucocorticoid therapy. The parameters that constructed the model should be easily obtained at most medical facilities. Meanwhile, the model should be able to stratify the death risks of HBV–ACLF patients received glucocorticoid treatment and find out the patients that are suitable to be treated with glucocorticoids.

## Materials and methods

### Patients and controls

A total of 280 HBV–ACLF patients were enrolled from July 2010 to June 2022, at Qilu Hospital of Shandong University. HBV–ACLF was diagnosed according to the consensus recommendations of APASL: (1) the presence of serum hepatitis B surface antigen (HBsAg) for at least 6 months; (2) progressive jaundice (serum total bilirubin ≥ 5 mg/dL); (3) coagulopathy (INR ≥ 1.5 or prothrombin activity < 40%) [1]. Exclusion criteria included: (1) severe psychiatric history, severe hypertension, active peptic ulcer, corneal ulcer, wound repair, epilepsy, severe diabetes, adrenal hyperfunction, and active tuberculosis (contraindications to glucocorticoid treatment) (2) uncontrolled infection or gastrointestinal hemorrhage before enrollment; (3) co-infected with human immunodeficiency virus (HIV), hepatitis A, C, D or E virus, Epstein–Barr virus, cytomegalovirus; (4) other liver diseases, such as alcoholic hepatitis, autoimmune liver diseases, etc. (5) decompensated liver cirrhosis; (6) liver cancer; (7) any other serious systemic disease that may interfere with the subject’s treatment or compliance, including serious renal, cardiac, respiratory, neurologic diseases, or other systemic diseases or tumors; (8) pregnancy.

### Therapeutic regimen

The conservative treatment for patients with HBV–ACLF was in accordance with the APASL consensus recommendations, including antiviral therapy with nucleoside analogs, absolute bed rest, intravenous infusion of albumin and plasma, energy supplements and vitamins, maintenance of water, electrolyte and acid–base equilibrium, prevention and treatment of complications, et al.

Among patients with HBV–ACLF, 204 patients received additional glucocorticoid treatment. Once the diagnosis of HBV–ACLF were confirmed, glucocorticoids (methylprednisolone or prednisolone) were administrated in these patients. To ensure comparable treatment effects, the dose of prednisone was converted to the equivalent dose of methylprednisolone on the basis of its anti-inflammatory potency. A dose of 1 mg/kg/d methylprednisolone was administered at D1–3. Then, the dose was gradually tapered off by 0.25 mg/kg/d during the remaining three 3-day cycles before withdrawn. Methylprednisolone 0.75 mg/kg/d was administered at D4–6, 0.5 mg/kg/d at D7–9, and 0.25 mg/kg/d at D10–12[10].

Informed consents were obtained from all participants. All co-authors had access to the study data and had reviewed and approved the final manuscript. The study protocol was approved by the local Research and Ethics Committee in Qilu Hospital of Shandong University, in accordance with the guidelines of the 1975 Declaration of Helsinki.

### Biochemical parameters collection

The peripheral blood of each patient was drawn on the first day of diagnosis. Hepatitis B surface antigen (HBsAg) and hepatitis B e antigen (HBeAg) were measured by an automatic analyzer (Cobas 6000 analyzer series, Roche Diagnostics, Basel, Switzerland). Serum biochemical markers (COBAS integra 800, Roche Diagnostics, Mannheim, Germany) included ALT, aspartate aminotransferase (AST), alkaline phosphatase (AKP), gamma-glutamyl transferase (GGT), albumin (ALB), total bilirubin (TBIL) and creatinine (Cr). HBV DNA level was measured by a real-time polymerase chain reaction (PCR) system (ABI 7300, Applied Biosystems, Foster City, CA, USA). Hemostasis markers (ACL TOP 700, Instrument laboratory, Lexington, MA, USA) included prothrombin time-international normalized ratio (INR). Hematological markers (Sysmex XE-2100, Sysmex Corporation, Japan) included white blood cell (WBC), hemoglobin (HGB) and platelet (PLT).

### Clinical parameters

Infection referred to existence of bacterial or fungal infection. Bacterial infection was diagnosed by a positive culture result and fungal infection was diagnosed according to EORTC/MSG definition [11]. Upper gastrointestinal hemorrhage (UGIH) was diagnosed according to the ACG Clinical Guideline [12]. Hepatic encephalopathy (HE) grades were reclassified into 0: non-HE, 1: mild (grades 1–2), and 2: severe (grades 3–4) according to the West-Haven criteria [13]. Ascites was detected by physical examination and abdominal ultrasound. We reclassified ascites grade into 0: no ascites, 1: mild, and 2: moderate to severe. Hepatorenal syndrome (HRS) was diagnosed according to the International Ascites Club’s guideline [14]. Electrolyte disturbance was defined as ≥ 1 electrolyte abnormalities of K ^+^ , Na ^+^ and Cl ^− ^. The presence of systemic inflammatory response syndrome (SIRS) was evaluated following the recommendations of the American College of Chest Physicians/Society of Critical Care Medicine Consensus Conference [15]. Child–Turcotte–Pugh (CTP) score, which included HE, prothrombin time (PT), ascites, TBIL, and serum albumin, was assessed according to the standard criteria [16]. MELD score was calculated according to the following formula:

*R* = 9.57 × log_e_[Cr (mg/dl)] + 3.78 × log_e_[TBIL (mg/dl)] + 11.2 × log_e_(INR) + 6.43 × (aetiology: 0 if cholestatic or alcoholic, 1 otherwise) [17].

### Follow-up of patients

The start date of follow-up was the date of the diagnosis of HBV–ACLF. All the patients were followed up for 90 days and the outcomes (death or survival) were recorded. The survival time was calculated from the date of the diagnosis to the date of death or the end of 90-day follow-up.

### Statistical analysis

Quantitative variables were expressed as median (centile 25; centile 75). Categorical variables were expressed as number (percentage). The data were analyzed using SPSS 16.0 statistical software (SPSS Inc., Chicago, IL, USA). Student’s *t* test and Mann–Whitney *U* test were used to compare quantitative variables. Chi-square test was used to compare categorical variables. In the training cohort, univariate cox proportional hazards regression analysis was performed to determine the association of demographic, biochemical, clinical parameters with 90-day mortality. Then, the variables with a *P* value of < 0.05 in the univariate regression analyses were introduced into a forward conditional stepwise cox proportional hazards regression to identify independent predictors. The conditional probabilities for stepwise entry and removal of a factor were 0.05 and 0.10, respectively. A prognostic index (PI = b1 × 1 + b2 × 2… + bnxn) was calculated.

The area under the receiver operating characteristic curve (AUC) was used to determine the predictive value of the prognostic index, MELD score and CTP score for 90-day mortality of HBV–ACLF. Sensitivity and specificity were used to identify the diagnostic accuracy. Survival curves were drawn with the Kaplan–Meier method and the statistical significances were determined by log-rank test. All statistical analyses were two sided. *P* < 0.05 was considered to be statistically significant.

## Results

### General characteristics

**Fig. 1:**
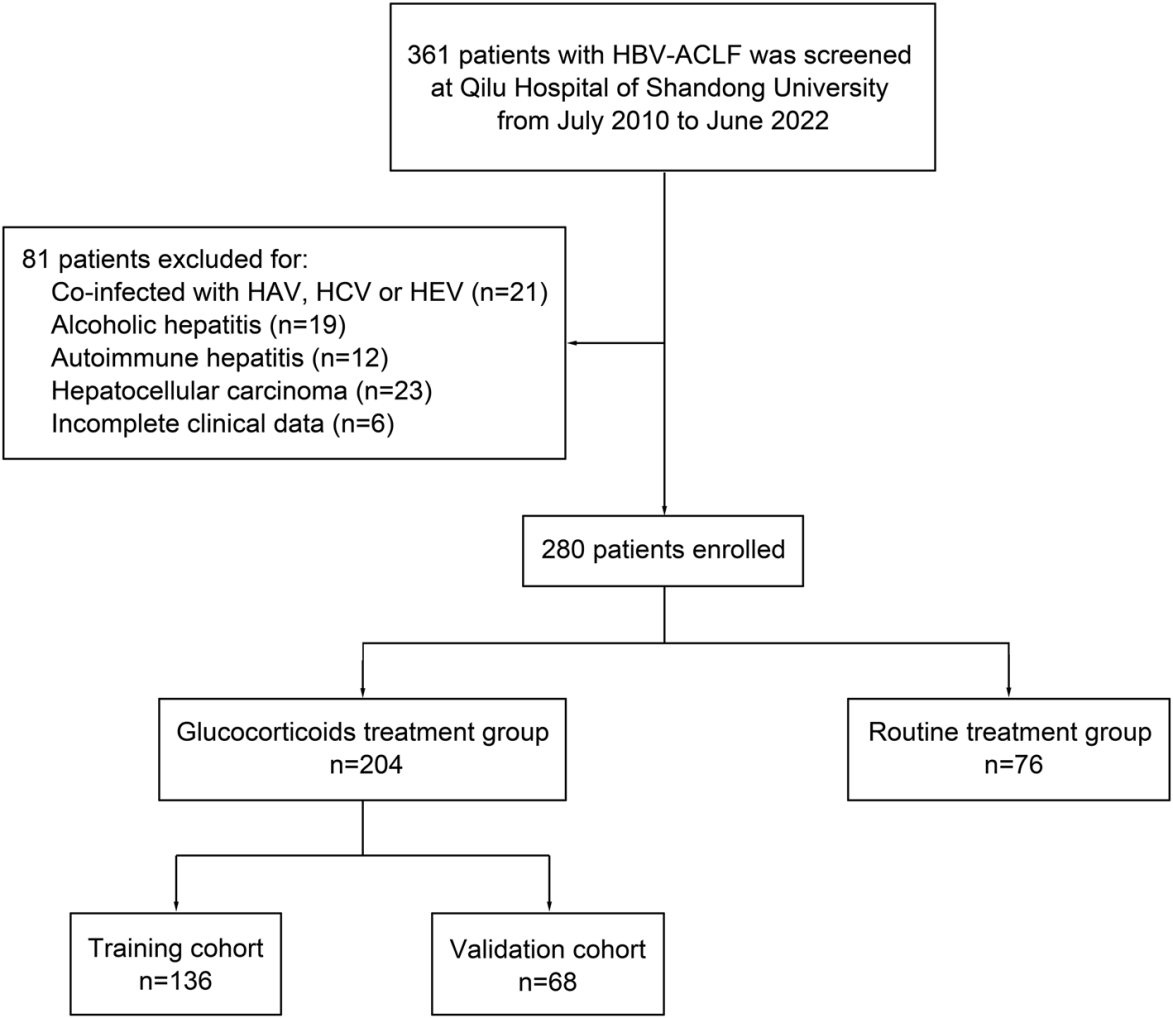
Flow diagram depicting the patients’ selection process

From July 2010 to June 2022, 361 patients with HBV–ACLF were screened at Qilu Hospital of Shandong University. Twenty-one patients with HBV–ACLF were excluded for co-infected with HAV, HCV or HEV. Nineteen patients were excluded for alcoholic hepatitis and 12 for autoimmune hepatitis. Twenty-three patients were excluded for hepatocellular carcinoma and 6 for incomplete clinical data. Finally, 280 patients were enrolled, among which 204 patients received additional glucocorticoid treatment (Fig. 1). Then, the patients received additional glucocorticoid treatment were randomly divided into a training cohort and a validation cohort. The training cohort included twice as many patients as the validation cohort [18]. Therefore, the training cohort included 136 patients and the validation cohort included 68 patients.

In this study, there were 7 patients in the glucocorticoid treatment group and 5 patients in the routine treatment group received liver transplantation. No significant difference was observed between them (*P* = 0.25). In the glucocorticoid treatment group, 3 patients in the training cohort and 4 patients in the validation cohort received liver transplantation (*P* = 0.17).

In this study, there were 66 (32.35%) patients in the glucocorticoid treatment group and 31 (40.79%) patients in the routine treatment group received artificial liver support (*P* = 0.19). Meanwhile, all the patients in this study received antiviral drugs. In the routine treatment group, 1 patient received lamivudine (LAM), 7 patients received adefovir dipivoxil (ADV), 30 patients received entecavir (ETV), 28 patients received tenofovir disoproxil fumarate (TDF) and 10 patients received tenofovir alafenamide fumarate (TAF). In the glucocorticoid treatment group, 4 patients received LAM, 19 patients received ADV, 89 patients received ETV, 65 patients received TDF and 27 patients received TAF. No significant difference was observed of the antiviral treatment strategy between the two groups (*P* = 0.94).

Table 1 presents the basic characteristics of the participants. There were no significant differences of the demographic, biochemical and clinical parameters between patients received additional glucocorticoid treatment and those not.

**Table 1:** Basic characteristics of patients with HBV–ACLF received glucocorticoid treatment or not

Variables	Routine treatment group (*n* = 76)	Glucocorticoid treatment group (*n* = 204)	*P*
Male sex, *n* (%)	58 (76.32%)	161 (78.92%)	0.64
Age (year)	38 (48–58)	35 (45–55)	0.1
HBeAg + n. (%)	46 (60.53%)	118 (57.84%)	0.69
Log_10_ [HBV DNA]	5.07 (4.21–6.23)	4.73 (3.38–6.1)	0.1
ALT (U/L)	210.5 (105.75–533)	172.5 (91–397.5)	0.34
AST (U/L)	176.5 (90.5–297.75)	158.5 (97–388.75)	0.64
GGT (U/L)	85.5 (53.25–145.75)	93 (59.5–153)	0.22
AKP (U/L)	130 (107.25–164.25)	142.50 (116–174.75)	0.17
TBIL (μmol/L)	232.9 (152.33–405.28)	276.70 (184.58–415.03)	0.17
ALB (g/L)	30.9 (26.75–34.5)	31.70 (29–34.78)	0.27
INR	1.88 (1.69–2.33)	1.82 (1.61–2.33)	0.28
Cr (μmol/L)	67 (53.25–80)	66 (57–76.75)	0.83
WBC (× 10^9^/L)	6.92 (4.45–10.22)	7.17 (4.73–9.34)	0.85
HGB (g/L)	125 (109–135.75)	125 (113–140)	0.86
PLT (× 10^9^/L)	99.5 (69–134.75)	109.00(67.25–154.75)	0.39
HE n. (%)	24 (31.58%)	62 (30.39%)	0.85
Ascites n. (%)	36 (47.39%)	104 (50.98%)	0.59
SIRS n. (%)	27 (35.53%)	64 (31.37%)	0.51
MELD score	21.52 (17.26–25.41)	21.55 (18.34–25.01)	0.78
CTP score	10 (9–11)	10 (9–11)	0.52
90-day mortality	45/76 (59.21%)	86/204 (42.16%)	0.01

Quantitative variables were expressed as median (centile 25; centile 75). Categorical variables were expressed as number

Baseline characteristics of the patients received glucocorticoid treatment are shown in Table 2. No significant differences of the demographic, biochemical and clinical parameters were observed between patients in the training cohort and validation cohort.

**Table 2:** Basic characteristics of patients received glucocorticoid treatment in the training and validation cohort

Variables	Training cohort (*n* = 136)	Validation cohort (*n* = 68)	*P* value
Male gender *n*. (%)	108 (79.41%)	53(77.94%)	0.81
Age (yr)	43.5(35.00–56.75)	46.0(37.00–54.00)	0.30
HBeAg + *n*. (%)	83 (61.03%)	35(51.47%)	0.19
Log_10_ [HBV DNA]	4.77(3.06–6.22)	4.61(3.64–5.95)	0.78
ALT (U/L)	198.00(91.00–387.50)	140.00(91.25–421.00)	0.54
AST (U/L)	169.00(97.50–417.75)	136.50(90.50–242.00)	0.07
GGT (U/L)	90.00(59.00–138.75)	109.50(61.75–180.25)	0.17
AKP (U/L)	142.50(116.00–173.75)	142.50(117.25–182.00)	0.95
TBIL (μmol/L)	302.95(184.58–417.18)	272.60(183.95–406.20)	0.27
ALB (g/L)	31.65(29.00–34.40)	32.45(29.30–36.05)	0.47
INR	1.76(1.56–2.11)	2.19(1.78–2.69)	< 0.01
Cr (μmol/L)	67.00(58.25–77.75)	65.00(53.50–73.75)	0.15
WBC (× 10^9^/L)	7.16(4.73–9.26)	7.17(4.42–9.70)	0.50
HGB (g/L)	125.00(114.25–140.00)	126.50(108.75–140.75	0.94
PLT (× 10^9^/L)	103.00(65.00–147.50)	123.50(73.75–165.00)	0.11
HE *n*. (%)	46 (33.82%)	16(23.53%)	0.13
Ascites *n*. (%)	71 (52.21%)	33(48.53%)	0.62
SIRS *n*. (%)	45 (33.09%)	19(27.94%)	0.46
MELD score	21.43(18.26–24.34)	22.20(18.47–26.66)	0.17
CTP score	10.00(9.00–11.00)	10.00(9.00–11.00)	0.25
90-day mortality	55/136 (40.44%)	31/68 (45.59%)	0.48

Quantitative variables were expressed as median (centile 25; centile 75). Categorical variables were expressed as number

### The treatment efficacy of glucocorticoids in HBV–ACLF

**Fig. 2:**
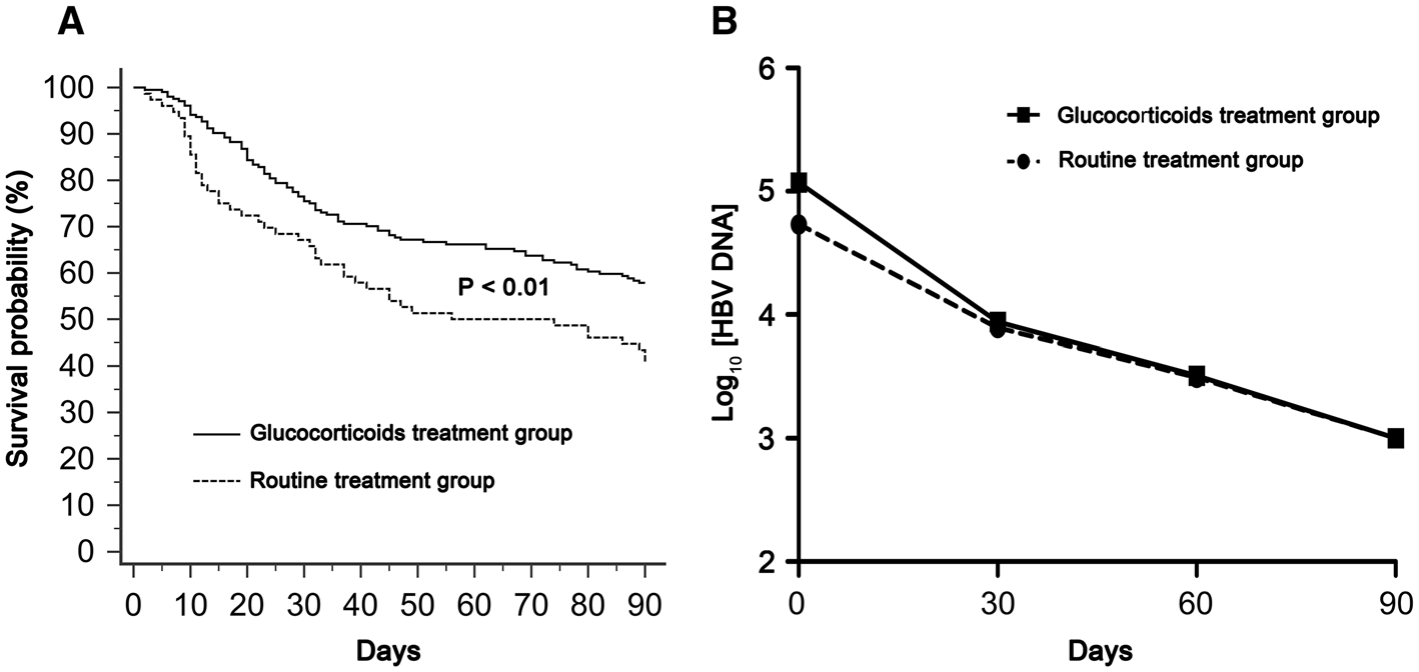
Treatment efficacy of glucocorticoids in HBV–ACLF. **A** Kaplan–Meier graph showing survival probability in patients with HBV–ACLF received additional glucocorticoid treatment or not. **B** Comparison of log_10_ [HBV DNA] between the glucocorticoid treatment group and routine treatment group

After 90-day follow-up, the mortality of patients in the glucocorticoid treatment group was 86/204 (42.16%), which was significantly lower than that of patients in the routine treatment group (45/76, 59.21%, *P* = 0.01). The mean survival time was 66.39 (SE 2.22, 95% CI 62.04–70.74) days in the patients received additional glucocorticoid treatment and 55.53 (SE 4.07, 95% CI 47.55–63.50) days in those not. Patients received additional glucocorticoid treatment showed significantly better survival that those not (*P* < 0.01, Fig. 2A).

There were no significant differences in log_10_ [HBV DNA] levels between the glucocorticoid treatment group (median 4.73, interquartile range 3.38–6.1) and routine treatment group (median 5.07, interquartile range 4.21–6.23) before treatment (*P* = 0.1, Table 1). Meanwhile, there were also no significant differences of log_10_ [HBV DNA] levels between the glucocorticoid treatment group and routine treatment group at 30 days (median 3.89, interquartile range 3.0–4.78 vs median 3.94, interquartile range 3.51–4.67, *P* = 0.38), 60 days (median 3.49, interquartile range 3.0–3.81 vs median 3.51, interquartile range 3.0–3.81, *P* = 0.32) and 90 days (median 3.0, interquartile range 2.7–3.48 vs median 3.0, interquartile range 2.7–3.08, *P* = 0.1) after treatment (Fig. 2B).

### The Adverse events of glucocorticoid treatment in HBV–ACLF

The incidence of newly onset infection in the glucocorticoid treatment group was 31.37% (64/204), which was higher than that in the routine treatment group (17/76, 22.37%). However, no significant difference was observed between them (*P* = 0.14).

The incidence of UGIH was 9.31% (19/204) in the glucocorticoid treatment group, whereas 7.89% (6/76) in the routine treatment group (*P* = 0.71). The incidence of HRS was 7.84% (16/204) in the glucocorticoid treatment group, whereas 10.53% (8/76) in the routine treatment group (*P* = 0.48). The incidence of electrolyte disturbance was 42.16% (86/204) in the glucocorticoid treatment group, whereas 39.47% (30/76) in the routine treatment group (*P* = 0.69).

### Predictors of 90-day mortality of HBV–ACLF after glucocorticoid treatment

Baseline demographic, clinical and laboratory parameters for the prediction of 90-day mortality in patients received additional glucocorticoid treatment were investigated by univariate analysis with cox proportional hazard regression model. In the training cohort, the univariate analysis identified eight variables including age (*P* = 0.002), TBIL (*P* < 0.001), ALB (*P* = 0.018), INR (*P* = 0.038), Cr (*P* = 0.013), HGB (*P* = 0.025), HE (*P* < 0.001), SIRS (*P* < 0.001) (Table 3). Then, these variables were introduced into a multivariate stepwise cox regression. HE (HR 1.940, 95% CI 1.379–2.728, *P* < 0.001), INR (HR 1.055, 95% CI 1.006–1.107, *P* = 0.027), TBIL (HR 1.003, 95% CI 1.002–1.005, *P* < 0.001), age (HR 1.031, 95% CI 1.010–1.052, *P* = 0.004) and SIRS (HR 3.312, 95% CI 1.855–5.913, *P* < 0.001) were identified to be independent predictors for 90-day mortality (Table 4).

**Table 3:** Factors associated with 90-day mortality in univariate analysis in the training cohort

Variables	Coefficient	HR	95% CI	*P* value
Gender	0.324	1.382	0.676–2.824	0.375
Age	0.033	1.034	1.012–1.056	0.002
HbeAg	0.005	1.005	0.583–1.732	0.985
Log_10_ [HBV DNA]	− 0.042	0.959	0.821–1.120	0.598
ALT	0.000	1.000	0.999–1.001	0.946
AST	0.000	1.000	0.999–1.001	0.558
GGT	− 0.002	0.998	0.994–1.001	0.189
AKP	0.000	1.000	0.997–1.003	0.924
TBIL	0.004	1.004	1.002–1.005	< 0.001
ALB	− 0.068	0.934	0.883–0.988	0.018
INR	0.043	1.044	1.002–1.088	0.038
Cr	0.015	1.015	1.003–1.026	0.013
WBC	0.015	1.015	0.952–1.084	0.643
HGB	− 0.013	0.987	0.975–0.998	0.025
PLT	− 0.003	0.997	0.992–1.002	0.193
HE	0.805	2.236	1.648–3.035	< 0.001
Ascites	− 0.085	0.919	0.616–1.370	0.678
SIRS	1.590	4.904	2.844–8.457	< 0.001

**Table 4:** Independent predictors for 90-day mortality identified after multivariate analysis in the training cohort

Variables	Coefficient	HR	95% CI	*P* value
HE	0.663	1.940	1.379–2.728	< 0.001
INR	0.054	1.055	1.006–1.107	0.027
TBIL	0.003	1.003	1.002–1.005	< 0.001
Age	0.03	1.031	1.010–1.052	0.004
SIRS	1.198	3.312	1.855–5.913	< 0.001

### Calculation of the prognostic index in the training cohort

A prognostic model was calculated by combining the 5 prognostic predictors with the regression coefficients reported in Table 4. It was calculated according to the following formula:

$${\text{HITAS score}} = \, 0.{663 } * {\text{ HE}} + \, 0.0{54 } * {\text{ INR }} + 0.00{3 } * {\text{ TBIL }} + \, 0.0{3 } * {\text{ Age }} + { 1}.{198 } * {\text{SIRS}}$$

### Diagnostic value of the HITAS score

**Fig. 3:**
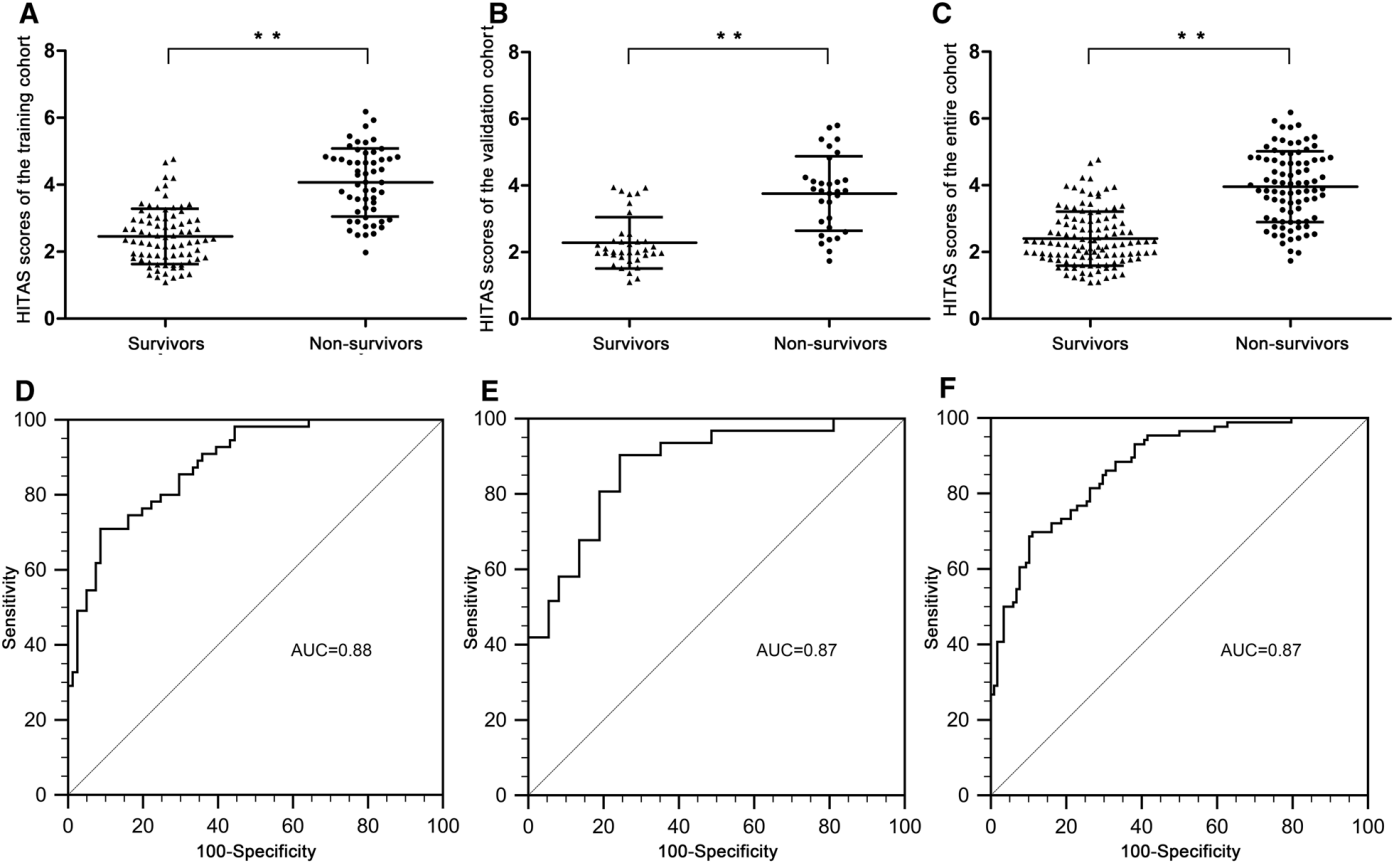
HITAS scores between survivors and non-survivors, and the ROC curves of HITAS scores. **A** HITAS scores of non-survivors were higher than those of survivors in the training cohort. **B** HITAS scores of non-survivors were higher than those of survivors in the validation cohort. **C** HITAS scores of non-survivors were higher than those of survivors in the entire cohort. **D** ROC curves of HITAS scores in the training group (AUC 0.88). **E** ROC curves of HITAS scores in the validation group (AUC 0.87). **F** ROC curves of HITAS scores in the entire cohort (AUC 0.87)

The HITAS scores of non-survivors were higher than those of survivors in the training cohort (*t* = − 9.754, *P* < 0.01) (Fig. 3A), the validation cohort (*t* = − 6.234, *P* < 0.01) (Fig. 3B) as well as the entire cohort (*t* = − 11.404, *P* < 0.01) (Fig. 3C). The AUC of the HITAS score was 0.88 (standard error [SE] 0.03, 95% confidence interval [CI] 0.82–0.93) in the training cohort (Fig. 3D), 0.87 (SE 0.04, 95% CI 0.77–0.94) (Fig. 3E) in the validation cohort and 0.87 (SE 0.02, 95% CI 0.82–0.92) (Fig. 3F) in the entire cohort.

In the training cohort, two cutoff points were chosen for HITAS score to predict the 90-day mortality in patients with HBV–ACLF received additional glucocorticoid treatment. A high cutoff point was chosen based on the ROC analysis of this model in the training cohort to provide a specificity of at least 85%. A low cutoff point was chosen to provide a sensitivity of at least 90% [19]. Finally, 2.5 was chosen as a low cutoff point and 3.47 was chosen as a high cutoff point. The diagnostic accuracy of HITAS score is presented in Table 5. In the training cohort, the cutoff of 2.5 provided a sensitivity of 95% and a specificity of 56%. The cutoff of 3.47 provided a sensitivity of 69% and a specificity of 91%, respectively. In the validation cohort, the cutoff point of 2.5 provided a sensitivity of 81% and a specificity of 76%. The cutoff point of 3.47 provided a sensitivity of 68% and a specificity of 84%, respectively.

**Table 5:** Diagnostic accuracy of the HITAS score in predicting the 90-day mortality of HBV–ACLF patients received glucocorticoid treatment.

Patients set	Cutoff points	No.	Survivors	Non-survivors	SE % (95% CI)	Sp % (95% CI)	PPV % (95% CI)	NPV % (95% CI)	PLR% (95% CI)	NLR% (95% CI)
Training cohort		136	81	55						
	≤ 2.5	48	45	3						
	> 2.5	88	36	52	95 (85–99)	56 (44–67)	59 (48–70)	94 (83–99)	2.1 (1.7–2.6)	0.1 (0.03–0.3)
	≤ 3.47	91	74	17						
	> 3.47	45	7	38	69 (55–81)	91 (83–97)	84 (71–94)	81 (72–89)	8.0 (6.6–9.7)	0.3 (0.2–0.8)
Validation cohort		68	37	31						
	≤ 2.5	34	28	6						
	> 2.5	34	9	25	81 (63–93)	76 (59–88)	74 (55–87)	82 (66–93)	3.3 (2.6–4.3)	0.3 (0.1–0.6)
	≤ 3.47	41	31	10						
	> 3.47	27	6	21	68(49–83)	84(71–95)	78(60–93)	76(61–88)	5.0(3.8–6.6)	0.4(0.1–1)

Using the cutoff points of 2.5 and 3.47, the HITAS score identified patients that have low (HITAS score ≤ 2.5), intermediate (2.5 < HITAS score ≤ 3.47), and high (HITAS score > 3.47) risks of death (Table 6). In the training cohort, only 3 of the 48 patients (6.25%) in the low-risk group died within 90 days. 14 of the 43 patients (32.56%) in the intermediate-risk group died. 38 of the 45 patients (84.44%) in the high-risk group died. In the validation cohort, only 6 of the 34 patients (17.65%) in the low-risk group died within 90 days. 4 of the 7 patients (57.14%) in the intermediate-risk group died. 21 of the 27 patients (77.78%) in the high-risk group died. In the entire cohort, only 9 of the 82 patients (10.98%) in the low-risk group died within 90 days. 18 of the 50 patients (36.00%) in the intermediate-risk group died. 59 of the 72 patients (81.94%) in the high-risk group died.

**Table 6:** Stratification of the risks of death by HITAS score in patients with HBV–ACLF received glucocorticoid treatment

Group	HITAS score	90-day mortality (%)		
		Training cohort %	Validation cohort %	Entire cohort %
Low-risk group	HITAS score ≤ 2.5	6.25	17.65	10.98
Intermediate-risk group	2.5 < HITAS score ≤ 3.47	32.56	57.14	36.00
High-risk group	HITAS score > 3.47	84.44	77.78	81.94

**Fig. 4:**
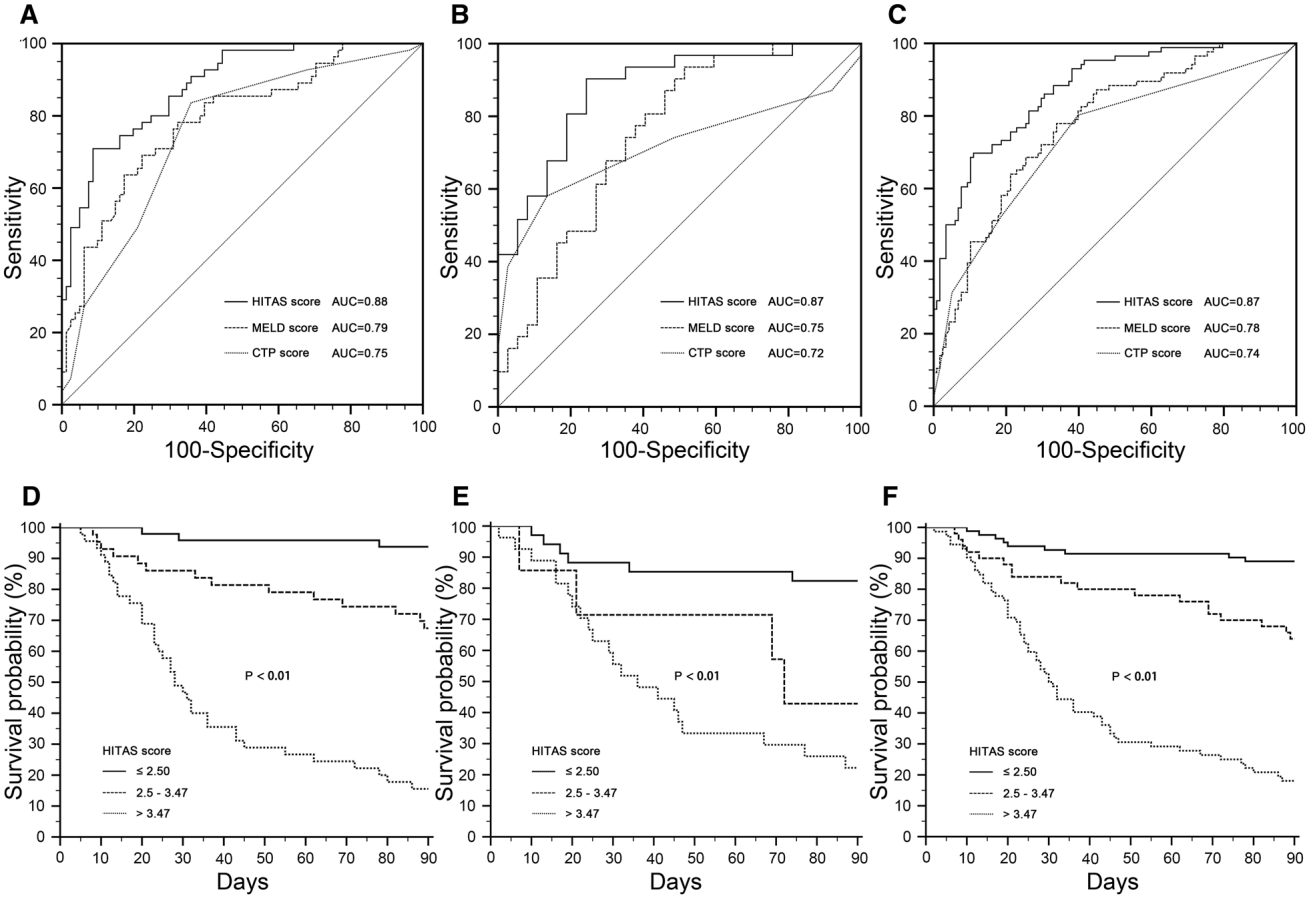
Receiver operating characteristic (ROC) curves and Kaplan–Meier graphs in patients with HBV–ACLF received glucocorticoid treatment. **A** Comparison among ROC curves of HITAS score*,* MELD score and CTP score in the training cohort. **B** Comparison among ROC curves of HITAS score*,* MELD score and CTP score in the validation cohort. **C** Comparison among ROC curves of HITAS score*,* MELD score and CTP score in the entire cohort. **D** Kaplan–Meier graphs showing survival probability in patients within the low-risk group, intermediate-risk group and high-risk group in the training cohort. **E** Kaplan–Meier graphs showing survival probability in patients within the low-risk group, intermediate-risk group and high-risk group in the validation cohort. **F** Kaplan–Meier graphs showing survival probability in patients within the low-risk group, intermediate-risk group and high-risk group in the entire cohort

In the training cohort (Fig. 4A), the AUC of HITAS score (AUC 0.88 SE 0.03, 95% CI 0.82–0.93) was significantly higher than MELD score (AUC 0.79 SE 0.04, 95% CI 0.71–0.85; *P* = 0.02) and CTP score (AUC 0.75 SE 0.04, 95% CI 0.67–0.82; *P* < 0.01). There was no significant difference between AUC of MELD score and CTP score (*P* = 0.50). In the validation cohort (Fig. 4B), the AUC of HITAS score (AUC 0.87 SE 0.04, 95% CI 0.77–0.94) was significantly higher than that of MELD score (AUC 0.75 SE 0.06, 95% CI 0.63–0.85; *P* = 0.04) and CTP score (AUC 0.72 SE 0.07, 95% CI 0.59–0.82; *P* = 0.02). No significant difference was found between AUC of MELD score and CTP score (*P* = 0.63). In the entire cohort (Fig. 4C), the AUC of HITAS score (AUC 0.87 SE 0.02, 95% CI 0.82–0.92) was significantly higher than that of MELD score (AUC 0.78 SE 0.03, 95% CI 0.71–0.83; *P* < 0.01) and CTP score (AUC 0.74 SE 0.06, 95% CI 0.68–0.80; *P* < 0.01). There was no significant difference between AUC of MELD score and CTP score (*P* = 0.40).

After 90-day follow-up, the mortality was 55/136 (40.44%) in the training cohort, 31/68 (45.59%) in the training cohort and 86/204 (42.16%) in the entire cohort. The mean survival time was 67.5 (SE 2.69, 95% CI 62.23–72.77) days in the training cohort, 64.16 (SE 3.92, 95% CI 56.49–71.84) days in the validation cohort and 66.39 (SE 2.22, 95% CI 62.04–70.74) days in the entire cohort. Then, we used Kaplan–Meier survival analyses to compare the survival probability in patients within the low-risk group, intermediate-risk group and high-risk group. As shown in Fig. 4D, E, F, the cutoff points of 2.5 and 3.47 successfully identified patients with low, intermediate, and high risks of death after 90-day follow-up in the training cohort, validation cohort and entire cohort (*P* < 0.01, respectively).

### Clinical application of the model

**Fig. 5:**
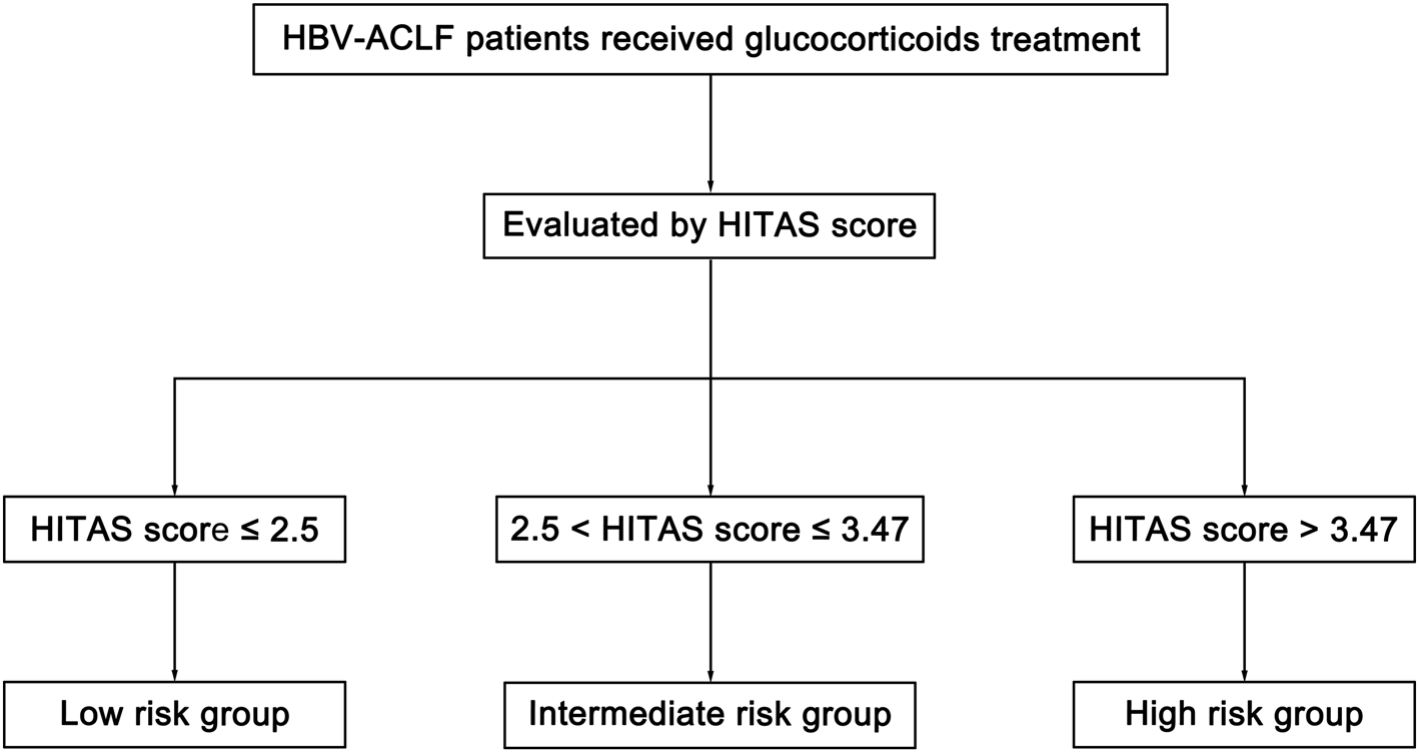
Algorithm for application of HITAS score to predict 90-day mortality in patients with HBV–ACLF received glucocorticoid therapy

Algorithm for the application of HITAS score to predict 90-day mortality of patients with HBV–ACLF received glucocorticoid treatment is presented in Fig. 5. The HITAS score is an early prediction model for the prognosis of HBV–ACLF, which might be used to identify HBV–ACLF patients with favorable responses to glucocorticoid treatment.

## Discussion

The short-term mortality of HBV–ACLF is extremely high. Until now, liver transplantation is still difficult to apply more widely in many countries due to the limitation of liver donors as well as the patients’ economic situation. Therefore, It is essential to develop more effective therapies for HBV–ACLF [20]. In this study, we demonstrated that glucocorticoid might have good efficacy in the treatment of HBV–ACLF. Patients received glucocorticoid treatment showed significantly better survival that those not. Meanwhile, we established a noninvasive model for predicting the 90-day mortality in patients with HBV–ACLF received glucocorticoid treatment. The HITAS score was constructed by five independent predictors: HE grade, INR, TBIL, age and SIRS status. It showed significantly better predictive value than the MELD score and CTP score. Using the cutoff points of 2.5 and 3.47, the HITAS score identified patients that have low, intermediate, and high risks of death. Therefore, it might be used to identify HBV–ACLF patients with favorable responses to glucocorticoid treatment.

HBV induced liver failure is caused by an overwhelming immune response against HBV [21]. Glucocorticoids, which are effective in suppressing the host–immune responses, have been identified to be beneficial in HBV–ACLF in many studies. However, there are still concerns over the use of glucocorticoid in HBV–ACLF until now. A big concern is that glucocorticoid may enhance HBV replication. In this study, the patients with HBV–ACLF also received nucleoside analogs to inhibit HBV replication combined with glucocorticoids. There were no significant differences of HBV DNA levels between the glucocorticoid treatment group and routine treatment group before treatment and at 30 days, 60 days and 90 days after treatment. Large number of studies have also demonstrated that the safety and efficacy of the combination therapy of glucocorticoids and nucleoside analogs. In the study performed by Yasui et al. the combination therapy of glucocorticoids and nucleoside analogs were effective in suppressing HBV replication in patients with HBV–ACLF, and a rapid decline of HBV DNA were found in survived patients[6]. Another study performed by Fujiwara et al. showed that HBV DNA decreased significantly during the 4-week period from the start of the glucocorticoids and nucleoside analogs therapy in severe acute exacerbation of chronic hepatitis B (CHB) [22].

In this study, HE was identified as an independent predictor for 90-day mortality in patients with HBV–ACLF received additional glucocorticoid treatment, which was consistent with several other studies. In a study performed by Romero-Gomez et al., HE was associated with a high mortality rate in a hospitalized cirrhotic patient and its presence added further to the mortality of patients with ACLF [23]. In several models established for predicting mortality of HBV–ACLF, HE was also identified as an independent risk factor [24, 25].

ACLF is mainly manifested by jaundice and coagulopathy. TBIL and INR are important markers of hepatocellular necrosis. In this study, TBIL and INR were also included in the HITAS score. In a previous systematic review, a detailed analysis of 19 studies and 73 prognostic indicators also showed that TBIL, INR appeared to be promising candidates for predicting poor prognosis in patients with ACLF [26].

SIRS and age were identified as independent risk factors in HITAS score. Glucocorticoids can suppress the host–immune response and promote the progression of severe infection. Therefore, it was reasonable that SIRS was associated with worse prognosis in patients with HBV–ACLF received additional glucocorticoid treatment. In a previous study, SIRS was found to be a major determinant of multiple organ failure and mortality in alcoholic hepatitis in the presence or absence of infections [27]. Older patients usually associated with longer duration of underlying diseases, higher frequency of comorbidity and poor hepatic regeneration in response to acute injury. CANONIC study investigators reported that age was a prognostic marker in cirrhotic patients with or without ACLF [28].

HITAS score is a model that can stratify the risks of death in patients with HBV–ACLF received glucocorticoid treatment. It may provide guidance for the clinical usage of glucocorticoids in HBV–ACLF. Meanwhile, the HITAS score was constructed by routine parameters (demographic, biochemical, clinical parameters) that could be easily obtained at admission. It is suitable to be applied in most medical facilities and allows a rapid identification of prognosis.

However, this study also has several limitations. First, the HITAS score was constructed in a training group and then validated in an independent validation cohort. The sample size of the validation cohort was relatively small. Further validation of HITAS score in a large cohort and prospective multi-center study is essential prior to its clinical application. Second, we only obtained variables at admission to construct the model. The data collection at different time intervals might provide more valuable information. Thirdly, the study was performed in a Chinese population. Further validation is still needed prior to its application in other regions of the world.

In conclusion, we constructed a non-invasive model to predict the 90-day mortality in patients with HBV–ACLF received glucocorticoid treatment. It can stratify the risks of death in patients with HBV–ACLF and might be used to identify HBV–ACLF patients with favorable responses to glucocorticoid treatment.

## Data Availability

The data underlying this article will be shared on reasonable request to the corresponding author.
